# On the importance of long-term functional assessment after stroke to improve translation from bench to bedside

**DOI:** 10.1186/2040-7378-3-6

**Published:** 2011-06-18

**Authors:** Thomas Freret, Pascale Schumann-Bard, Michel Boulouard, Valentine Bouet

**Affiliations:** 1 GMPc - Groupe Mémoire et Plasticité comportementale, EA4259, Université de Caen Basse-Normandie, Caen, France

## Abstract

Despite extensive research efforts in the field of cerebral ischemia, numerous disappointments came from the translational step. Even if experimental studies showed a large number of promising drugs, most of them failed to be efficient in clinical trials. Based on these reports, factors that play a significant role in causing outcome differences between animal experiments and clinical trials have been identified; and latest works in the field have tried to discard them in order to improve the scope of the results. Nevertheless, efforts must be maintained, especially for long-term functional evaluations. As observed in clinical practice, animals display a large degree of spontaneous recovery after stroke. The neurological impairment, assessed by basic items, typically disappears during the firsts week following stroke in rodents. On the contrary, more demanding sensorimotor and cognitive tasks underline other deficits, which are usually long-lasting. Unfortunately, studies addressing such behavioral impairments are less abundant. Because the characterization of long-term functional recovery is critical for evaluating the efficacy of potential therapeutic agents in experimental strokes, behavioral tests that proved sensitive enough to detect long-term deficits are reported here. And since the ultimate goal of any stroke therapy is the restoration of normal function, an objective appraisal of the behavioral deficits should be done.

## Letter to the editor

Regarding functional evaluations, the first point to consider is the body weight. Monitoring body weight changes after a stroke is of prime importance since postoperative weight loss may indicate feeding difficulties. Aside from all ethical considerations, such postoperative weight loss has been shown by some authors to be correlated with the extent of the lesion (extensive corticostriatal damage [1], or the involvement of the external carotid artery territory in the lesion [2]). An easy measurement such as this can advise as to the severity of the lesion. Beyond the lesion *per se*, feeding difficulties may also result from a reduced consciousness level or poor mobility due to anesthesia and/or surgery. For example, a surgical approach in which the temporal muscle is injured, such as the Tamura model [3] may induce severe mastication impairments, resulting thus in higher body weight loss. Nevertheless, a poor nutritional intake (Dennis, 2000) can be a bias, since it has been shown in patients [2] as in animals to have a negative effect on functional outcomes after stroke [2]. Body weight monitoring of patients has even been recommended as an index of functional outcome [4]. Thus, investigating weight changes in preclinical studies has to be recommended for all authors in the field, since it gives an independent and unambiguous assessment of animal welfare and safety. Animals should be weighed at least once before surgery and then regularly after. This parameter, accessible to everyone and not only to behaviorists since it does not require any specific skill, can also give, in some ways, information on how animals recover from surgery and can even be a prognostic index for functional outcome.

Concerning functional evaluations, few studies consider crucial long-term evaluation, even though it has been highly recommended during the Stroke Therapy Academic Industry Roundtables [5-7]. As in clinical practice [8], animals display a large degree of spontaneous recovery within a short time after experimental cerebral ischemia [9-12]. Even though demanding sensorimotor and cognitive tasks are powerful in revealing tiny deficits, long-term studies addressing such behaviors are unfortunately not very abundant. The characterization of long-term functional recovery is critical for evaluating the efficacy of potential therapeutic agents in an experimental stroke. Both acute (few days) and long-term (several weeks or months) evaluations have to be addressed in order to demonstrate a stable neuroprotection, and not only a slowing down of the lesion evolution [13,14]. The issue of including behavioral assessments in animal stroke studies becomes even more critical with the recent interest in neurorestorative strategies, which requires a longer period of administration than classical treatments. Effectiveness of such strategies is more likely observable *via *changes in synapse number and dendritic structure, for example, than by changes in infarct volume [15,16]. Since the ultimate goal of any stroke therapy is the restoration of functions that allow for a normal daily life of patients; an objective appraisal of the behavioral deficits should be done. Stroke-induced functional impairments can be divided into acute (pointing out effects of drugs on the rate of recovery - days or weeks) and long-term (pointing out the effects of drugs on the extent of recovery - several weeks to months). Ideally, a set of several different tests has to be performed to gather complementary information (see table 1: behavioral tests and time-points are given as an indication, and need to be adjusted according to the species/strain and the stroke model used). Several behavioral tests have been applied to ischemia research in regards to clinical criteria, from the simplest which measures global neurological status or motor reflexes (*i.e*. neurological score [17], limb placing test [18], cylinder test [19] - useful to assess an acute phase after stroke) to more complex tests assessing sensory and motor functions (*i.e*. adhesive removal [20] and rotarod or staircase [19,21-25]) - that are more relevant for the long-term phase. Similarly, cognitive tests such as those assessing memory functions are preferentially used for later time points because they require normalized motor functions [19,22,26-28]. These behavioral tests have to be carefully chosen in accordance with the drugs tested and the nature of the targeted cerebral structures. Except from that of Winter and colleagues [27], very few publications deal with stoke-induced disturbances in emotional behavior. Because anatomical and functional brain regions are affected on a different timescale, and because treatments may also differentially affect those regions, it is our view and that of others [29] that direct (cortex, striatum) or indirect (thalamus) anatomical substrates hit during stroke, rather than global brain lesions, may be critical determinants of behavioral impairments and outcome.

**Table 1:** Available and helpful sensorimotor tests assessing longitudinal and long-term functional recovery.

	Behavioral test	Brief description	Time points	Advantages	Concerns
SENSORI-MOTOR FUNCTIONS	Neurological scales	**Neurological score **for 4 to 6 items (ranking from normal motor function: *spontaneous walk initiation, circling behavior; *to normal posture at rest: *limb slipping arms, head tilting, hand crossing the chest*; or when lifted by the tail for rodents only: *flexion of torso and controlateral forelimb, decreased of controlateral forelimb grip*).	Few days to a week[33]	* Inter-species comparisons: non-human primate and rodents (mouse, rat, gerbil) * Useful to access the acute phase of cerebral ischemia	* Requires animal contention * Highly variable from a lab to another according to the number of evaluation criteria included and to the procedure which is highly experimenter-dependant
			
	Limb placing test	**Sensorimotor and proprioceptive abilities: **sensorimotor responses of fore- and hindlimbs to tactile, visual, and proprioceptive stimuli.	Till 2 - 3 weeks[34]		
	
	Cylinder test	**Limb-use asymmetry: **preference for using the non-impaired forelimb for weight shifting movement. Animal is placed in a cylinder and limb use asymmetry is observed during rearing with support.	Few weeks[35,36]	* Easy to perform * No need of animal contention	* Cannot be done in the acute phase, since it needs a certain level of recovery
	
	Grip strength test	**Muscular strength: **forelimb muscular strength with a Newton meter attached to a triangular steel wire grasped by the animal.	Few weeks to a month[37]	* Quantitatively measured by a Newton meter	* Specific apparatus required
	
	Beam walking test	**Locomotor function: **evaluation of forelimb and hindlimb faults while traversing along a ledged tapered beam.	Few weeks[38,39]	* Easy to perform * No need of expensive materials	* Cannot be done until postural bias and circling behaviors have not disappeared
	
	Rotarod test (constant or accelerated)	**Balance and motor coordination: **measure of latency to fall off a rotating rod (speed of rotation can be constant or increasing)	weeks[40,41]	* Easy to perform * Quantitative measures	* Cost of apparatus * May require a training session
	
	Adhesive removal test	**Somatosensory and motor function: **measure of the requested time to sense and to remove the adhesives placed on the animal's body (forelimb, hindlimb or snout). Of note, performance at this task has been shown to be strictly independent of postural bias and circling behaviors	From weeks[22] to months[42,43]	* Inter-species comparisons: non-human primate and rodents (mouse, rat, gerbil)	* May require a training session * Requires animal contention
	
	Reach to grasp test/Skilled reaching test/staircase test	**Forelimb ability and dexterity: **measure of the ability to reach food pellets	From weeks[44,45] to months[43]	* Inter-species comparisons: non-human primate and rodents (mice, rat) * independent forelimb reaching ability	* Requires a food restriction * Time-consuming * May require a training session

MNESIC FUNCTIONS	Morris water maze	**Spatial memory task: **Measure of the required distance and time, to get to a escape platform, hidden under the surface of the water in a circular pool tank	weeks[46]	* Highly develop since 80's, numerous protocols existing that can fit all request	* Require a training session for the learning phase and a retention phase * Require a dedicated room to lodge the pool * Require a software for the tracking of the animal
	
	Passive avoidance	**Fear-motivated task: **animal learns to refrain from stepping through a door to an apparently safer but previously punished dark compartment	From days[47] to weeks[46]	* One-trial task with no need to learn a rule	* Require electrical foot shock that may interfere with other behavioral test
	
	Object recognition test	**Non-spatial memory task: **Measure of spontaneous tendency of rodents to spend more time exploring a novel object than a familiar one.	weeks[48]	* One-trial task with no need to learn a rule	

EMOTIONAL-TRAIT	Elevated plus maze	**Anxiety-related behaviour: **measure of the time spent in anxiogenous (open) *vs *safe (close) arms of a labyrinth	Few months[27]	* Easy to perform * Inter-species comparison (mouse, rat)	* Cannot be done until postural bias and circling behaviors have not disappeared
	
	Black & white box	**Anxiety-related behaviour: **measure of the preference to stay in a dark *vs *illuminated compartment of a double-box			

Time points when behavioral tests can discern sham-operated from stroked animals are given as an indication for a typical 30-50% lesion size.

Whereas the correlation between acute histological lesions and early behavioral impairment is well documented[24], less is known about the long-term evolution of this relationship. Correlation studies have to take into account the different brain structures, primarily or secondarily affected by stroke, in order to bring a better understanding of their involvement in behavioral impairments [23,25,30]. Additionally, the development of non-invasive methods (such as MRI) allowing longitudinal assessment of the evolution of the lesion may bring new insights to understanding the mechanisms underlying spontaneous functional recovery [31]. Longitudinal correlations must then be promoted since therapeutic agents targeting those mechanisms will free us from the current mandatory 3 to 6 hour therapeutic window.

The use of clinically relevant models taking into account associated factors and/or pathologies (*i.e*. aging[32], arterial hypertension, diabetes, ..) have, moreover, to be reinforced in the incoming studies. As such, the post-ischemic recovery may be different, depending on the presence of these aggravating factors.

## Conflict of interests

The authors declare that they have no competing interests.
